# Association of Environmental Arsenic Exposure, Genetic Polymorphisms of Susceptible Genes, and Skin Cancers in Taiwan

**DOI:** 10.1155/2015/892579

**Published:** 2015-07-29

**Authors:** Ling-I Hsu, Meei-Maan Wu, Yuan-Hung Wang, Cheng-Yeh Lee, Tse-Yen Yang, Bo-Yu Hsiao, Chien-Jen Chen

**Affiliations:** ^1^Genomics Research Center, Academia Sinica, No. 128 Academia Road, Section 2, Nankang, Taipei 115, Taiwan; ^2^Graduate Institute of Oncology, College of Medicine, National Taiwan University, Taipei 100, Taiwan; ^3^School of Public Health, College of Public Health and Nutrition, Taipei Medical University, 250 Wu-Hsing Street, Taipei 110, Taiwan; ^4^Department of Medical Research, Shuang Ho Hospital, Taipei Medical University, No. 291 Zhongzheng Road, Zhonghe District, New Taipei City 23561, Taiwan; ^5^Graduate Institute of Clinical Medicine, College of Medicine, Taipei Medical University, 250 Wu-Hsing Street, Taipei 110, Taiwan; ^6^Molecular and Genomic Epidemiology Center, China Medical University Hospital, China Medical University, Taichung 404, Taiwan; ^7^Graduate Institute of Epidemiology, College of Public Health, National Taiwan University, 17 Xu-Zhou Road, Taipei 100, Taiwan

## Abstract

Deficiency in the capability of xenobiotic detoxification and arsenic methylation may be correlated with individual susceptibility to arsenic-related skin cancers. We hypothesized that glutathione S-transferase (GST M1, T1, and P1), reactive oxygen species (ROS) related metabolic genes (NQO1, EPHX1, and HO-1), and DNA repair genes (XRCC1, XPD, hOGG1, and ATM) together may play a role in arsenic-induced skin carcinogenesis. We conducted a case-control study consisting of 70 pathologically confirmed skin cancer patients and 210 age and gender matched participants with genotyping of 12 selected polymorphisms. The skin cancer risks were estimated by odds ratio (OR) and 95% confidence interval (CI) using logistic regression. EPHX1 Tyr113His, XPD C156A, and GSTT1 null genotypes were associated with skin cancer risk (OR = 2.99, 95% CI = 1.01–8.83; OR = 2.04, 95% CI = 0.99–4.27; OR = 1.74, 95% CI = 1.00–3.02, resp.). However, none of these polymorphisms showed significant association after considering arsenic exposure status. Individuals carrying three risk polymorphisms of EPHX1 Tyr113His, XPD C156A, and GSTs presented a 400% increased skin cancer risk when compared to those with less than or equal to one polymorphism. In conclusion, GSTs, EPHX1, and XPD are potential genetic factors for arsenic-induced skin cancers. The roles of these genes for arsenic-induced skin carcinogenesis need to be further evaluated.

## 1. Introduction

Arsenic (As) is a widely distributed element in the environment that is mainly transported by water. The arsenic species in polluted water are the inorganic trivalent form (arsenite) and the pentavalent form (arsenate); the trivalent form exhibits higher toxicity than does the pentavalent form. Arsenic has been associated with cardiovascular diseases, diabetes, neurological manifestations, and many cancers such as those of skin, bladder, liver, lung, kidney, and prostate [1–3]. Cumulative, long-term exposure to arsenic could cause dose-dependent, nonspecific chronic effects in the organs. Although the mechanism is not yet clear, arsenic compounds are designated as carcinogens by the International Agency for Research on Cancer (IARC).

Skin cancer is one of the most common arsenic-induced cancers. Among the types of skin cancers, squamous cell carcinoma and basal cell carcinoma, but not melanoma, are associated with the presence of arsenic in drinking water [4]. Previous studies showed that arsenic may contribute to the progression of skin cancer in association with carcinogen partner such as sunlight. A strong dose-response relationship between arsenic exposure and skin lesions has been consistently observed [5]. However, even among people with a relatively high arsenic exposure for many years, less than 30% of exposed victims were affected with skin lesions in Taiwan [6]. Genetic factors, nutritional status, ethnicity, an early age of exposure and variations in arsenic biotransformation are all potentially responsible for differences in individual susceptibility to arsenic-induced skin carcinogenesis.

Reduction of arsenic is associated with the production of reactive oxygen species (ROS) [7]. ROS such as hydrogen peroxide and hydroxyl radical could directly or indirectly degrade cellular compounds. Arsenic has been demonstrated to induce ROS formation in many cell types and thus cause DNA damage, protein modification, and lipid peroxidation in these cells [8]. An excess of ROS plays a role for certain cancer development. The theory that arsenic induces ROS to cause carcinogenicity could partially explain the high incidence of lung, bladder, and skin cancers [9].

Heme oxygenase (HO-1) and NAD(P)H:quinone oxidoreductase 1 (NQO1) are genes involved in the reduction of iron-mediated ROS. Increased oxidative stress will promote nuclear factor erythroid 2-related factor 2 (Nrf2) accumulation, which activates transcription of HO-1 and NQO-1. HO-1 catalyzes the rate-limiting step in heme degradation and is rapidly upregulated by ROS or heavy metals. The numbers of (GT)n repeats in promoter region are inversely related to its enzyme activity. Our previous studies have shown that shorter (GT)n repeats of HO-1 were associated with reduced arsenic-associated carotid atherosclerosis and cardiovascular mortality [10, 11]. One meta-analysis suggests that longer HO-1 (GT)n repeats are potential genetic factors for developing squamous cell carcinoma [12]. NQO1 has the multiple roles in the protection against cancer development including superoxide scavenging, quinones detoxification, tumor suppressor proteins stabilization, and microtubules stabilization. The C609T (Pro187Ser) polymorphism has been shown to have strong impact on gene product stability and enzyme activity. One comprehensive meta-analysis involving 92 studies suggests that C609T is an important genetic factor for overall cancer risk as well as bladder and gastric cancer risk [13]. Microsomal epoxide hydrolase (EPHX) is responsible for the hydrolysis of various epoxides. EPHX breaks the three-membered epoxide ring structure to form a less-reactive diol that can be more readily excreted. The T337G Tyr113His and A415G His139Arg polymorphisms have been reported associated with 40% decreased and 25% increased enzyme activity [14].

DNA repair is essential for maintaining genomic stability. Polymorphisms in DNA repair genes may result in differential DNA repair activity and further contribute to individual susceptibility for various cancer developments. Base excision repair (BER) is responsible for repairing oxidative DNA damage and single-strand breaks. The X-ray repair cross complementing group 1 (XRCC1) gene is involved in the BER pathway. Several SNPs in the XRCC1 have been reported for amino acid changes, including Arg399Gln G>A and Arg280His G>A. Several epidemiological studies and one meta-analysis suggest that these two SNPs are associated with the risk of nonmelanoma skin cancer [15–17]. Human 8-oxoguanine DNA* N*-glycosylase 1(*hOGG1*), also involved in BER pathway, is responsible for repairing DNA damage 8-oxo-7,8-dihydroguanine. There is a C>G transversion in exon 7, resulting in an amino acid change from serine to cysteine (Ser326Cys). The variant of this SNP is associated with an impairment of DNA glycosylase activity [18]. hOGG1 Ser326Cys polymorphism has been reported associated with various types of malignancy including lung, esophageal, bladder, and prostate cancer [19–21].

Nucleotide excision repair (NER) is responsible for the removal of UV protoproducts and bulky adducts. Xeroderma pigmentosum (XP) patients, deficient in the NER, have a strikingly increased (>1000-fold) risk of skin cancer. Xeroderma pigmentosum group D (*XPD*) involved in NER and mutations in the gene leads to NER defect [22]. It has been consistently documented that nonsynonymous polymorphism at XPD codon 156 is associated with NMSC [23, 24]. One study has also shown that XPD polymorphisms modify the association between arsenic exposure and NMSC [25].

In the present study, we aimed to examine the association of potential genetic factors with arsenic-induced skin cancers. This hospital-based case control study was conducted in southwestern Taiwan where high arsenic exposure was found in the drinking water. The polymorphisms of interests were classified into three groups according to their functions: (i) xenobiotic metabolic genes: glutathione S-transferases (GSTs) M1, T1, and P1 Ile105Val (rs1695); (ii) reactive oxidative stress (ROS) related genes: NQO1 C609T (rs1800566), EPHX1 Tyr113His (rs1051740) and His139Arg (rs2234922), and HO-1 5′-franking region GT repeats; (iii) DNA repair genes: XRCC1 Arg280His (rs25489) and Arg399Gln (rs25487), XPD C156A (rs238406), hOGG1 Ser326Cys (rs1052133), and ataxia-telangiectasia mutated (ATM) IVS48 +238C>G (rs609429).

## 2. Materials and Methods

### 2.1. Study Population

The study population was chosen as previous studies [11, 12]. Three villages, Ho-Mei, Fu-Hsing, and Hsing-Ming, of Putai Township along the southwest coast of Taiwan were selected as the study area. The names and addresses of all adult residents in the three villages under study were obtained from census records at the local census registration office, where sociodemographic characteristics including birth date, gender, marital status, educational level, and occupation of all the members of every household were registered and updated annually. From a total of 2258 residents aged 30 years or above who were registered within the study area, only 1571 individuals who resided at least 5 days a week in the village were recruited into the study. All the subjects were of the same Ming-Nang ethnicity. Between 1989 and 1996, about 1081 study subjects underwent six community health examinations. At this time, biological specimens were collected including blood buffy coat. About 30% of subjects refused to give the blood samples or had inadequate DNA samples. There were no differences in characteristics between the subjects with and without blood samples based on age, gender, educational level, and smoking status. A total of 764 (71%) adequate lymphocyte DNA samples were available for future study. The skin lesions of all participants were clinically diagnosed by the experienced dermatologists from Kaohsiung Medical University. Three age (±5) and gender matched controls for each case were selected from the study subjects with DNA available and with no history of any malignancy. Finally, a total of 70 pathological-confirmed skin cancer patients, including 57 patients with Bowen's diseases, 4 with basal cell carcinoma (BCC), 5 with squamous cell carcinoma (SCC), and 4 with Bowen's disease and BCC, and 210 age and gender matched controls were recruited for this study.

Each study participant was personally interviewed by a public health nurse. Information obtained from the interview included the history of drinking high arsenic artesian water, residential history, sociodemographic characteristics, lifestyle variables such as alcohol consumption, cigarette smoking, and dietary consumption frequency, and personal and family medical histories. The cumulative arsenic exposure index in ppm-year was defined as the sum of products by multiplying the arsenic concentration in well water by the duration of water consumption for consecutive periods of time as resident in each of the villages. This study was approved by the Institutional Review Board of the National Taiwan University College of Public Health, and the informed consent of all participants was obtained.

### 2.2. DNA Extraction and Genotyping

Buffy coat was isolated from 10 mL of blood using heparin and stored at −70°C until genomic DNA was extracted according to the manufacturer's protocol. All genetic polymorphisms of candidate genes, except GST M1, T1, and HO-1, were detected by polymerase chain reaction and restriction fragment length polymorphism (PCR-RFLP) analysis. The restriction enzymes used in the RFLP analysis are shown in Table 1. In brief, PCR products were digested by the restriction enzyme overnight and then separated on 2% agarose gels. The primers used to detect polymorphisms of GST M1, T1, and HO-1 are also shown in Table 1. PCR analysis was carried out in 2 *μ*L of diluted DNA, 1 *μ*L of each primer at 5 *μ*M, 1 *μ*L of 2.5 mM dNTPs, and 0.16 units of Taq polymerase. Cycling conditions are as follows: denaturation at 94°C for 4 minutes, 35 cycles of denaturation at 94°C for 40 seconds, annealing at 55°C for 30 seconds, elongation at 72°C for 40 seconds, and finally 72°C for 10 minutes as the last step. Null types of GST M1 and T1 would not have produced amplified products, whereas nonnull types would generate the PCR products. Thus, the different types of these two polymorphisms could be identified. The 5′-site of the HO-1 forward primer was labeled with FAM fluorescence dye in Table 1. PCR products were sequenced using the DNA sequencer (ABI prism 377) and then analyzed with the GeneScan software (Applied Biosystems, Foster City, CA, USA). The number of (GT)n in the HO-1 gene with more than 29 repeats was identified as long (L), and those with less than 28 repeats were identified as short (S).

### 2.3. Statistical Analysis

Distribution of cumulative arsenic exposure and monomethylarsonic acid (MMA) percentage between cases and controls was compared using the chi-square (*χ* ^2^) test. Genotypic frequencies were estimated by direct counting and *χ* ^2^ tests were utilized to determine the significance of the association between groups with a contingency table. The risk for skin cancers associated with the genotypes was estimated as an odds ratio (OR) and 95% confidence interval (CI) by logistic regression with adjustment for the effect of possible confounders such as age, gender, cumulative arsenic exposure, and MMA percentage. We performed gene-gene interaction separately for (1) the polymorphisms in GSTs family; (2) reactive oxidative stress related polymorphisms; (3) DNA repair related polymorphisms; and (4) the combination of the polymorphisms which showed significant association with the disease based on univariate analysis. All statistical tests were performed using SAS statistical software (version 9.3, SAS Institute Inc., Cary, NC, USA) on two-sided probabilities.

## 3. Results

The mean of age and the distributions of gender, cumulative arsenic exposure, and MMA percentage were presented in Table 2. Age and gender was not different among cases and controls. High cumulative arsenic exposure and high MMA percentage were associated with an increased skin cancer risk. Odds ratios (95% CIs) were 1.00 (referent), 3.55 (1.14–11.06), and 5.25 (1.72–16.05) for cumulative arsenic concentrations of <10.0, 10.0–19.9, 20.0+ ppm-years, respectively. *P* value for trend test was 0.0028. The subjects with higher MMA percentage (>13.81%) had a 101% increased disease risk when compared to the ones with lower percentage (OR = 2.01, 95% CI: 1.09–3.73).

In the univariate analysis of genetic polymorphisms (Table 3), GSTT1, EPHX1 exon 3, and XPD exon 6 showed significantly higher risks. The OR of GSTT1 null genotype was 1.74 (1.00–3.02). The ORs of EPHX1 exon 3 T/C and C/C polymorphisms were 3.74 (1.20–11.66) and 2.58 (0.85–7.85), respectively. When the T/C and C/C groups were combined, the OR compared with the T/T group was 2.99 (1.01–8.83). In addition, the ORs of A/C and A/A polymorphisms in XPD exon 6 were found to be 1.96 (0.91–4.22) and 2.24 (0.93–5.40), though the difference was not statistically significant. Statistical significance at borderline level was observed after the two groups were combined, with an OR of 2.04 (0.99–4.27). However, none of the polymorphisms in this study showed significant association with the disease if multiple comparison issue was considered.

In the multivariate analysis adjusted for age, gender, cumulative arsenic exposure, and MMA percentage, all the OR values were reduced (Table 4). No significant differences were found in EPHX1 exon 3, XPD exon 6, and GSTT1 polymorphism, although the ORs in the polymorphism groups remained higher than reference groups.

Subsequently, we performed gene-gene interaction separately for different groups with the adjustment for age, gender, cumulative arsenic exposure, and MMA percentage (Table 5). Among the xenobiotic metabolic genes (GST family), ORs in the 1-, 2-, and 3-polymorphism groups as compared to the 0-polymorphism group were 4.21 (1.38–12.85), 4.61 (1.52–13.98), and 3.58 (1.00–12.79), respectively (data not shown). When more than or equal to one polymorphism group was combined, the OR became 4.27 (1.48–12.38). Among the ROS-related metabolic genes, we chose NQO1, HO-1, and EPHX1 exon 3. In comparison to the referent group with less than two polymorphisms, ORs of the 2- and 3-polymorphism groups were 1.68 (0.86–3.26) and 1.88 (0.69–5.13), respectively. The *P* values of the two groups did not demonstrate significance. In the analysis of polymorphisms in DNA repair genes, we chose XPD, hOGG1, ATM, and XRCC1 exon 9. No significant differences were found in the 3- and 4-polymorphism groups compared to the group with less than 3 polymorphisms; the ORs were 4.40 (0.57–34.26) and 6.95 (0.86–56.23), respectively. However, the *P* value for trend was statistically significant (*P* = 0.0334).

Finally, we investigated the gene-gene interactions of GST genes, EPHX1 exon 3, and XPD exon 6, which showed significant differences between cases and controls in univariate analysis. When the groups with less than 2 polymorphisms were combined as a reference, the ORs of the 2- and 3-polymorphism groups were 1.56 (0.32–7.45) and 4.86 (1.08–21.85), respectively, while the *P* value for the trend was 0.0004. Moreover, we found significantly higher disease risk among the 3-polymorphism group, with a *P* value of 0.0392.

## 4. Discussion

In the present study, we analyzed 12 polymorphisms in 10 genes in an established population in Taiwan. A total of 70 cases and 210 healthy control individuals were included in this study. Similar to previous studies, the subpopulation with higher cumulative arsenic exposure had a higher risk in developing arsenic-induced skin cancer [26–28]. Although the group having a high MMA percentage also had a higher OR, the data was not completely comparable to former case control studies on arsenic-induced skin cancer. In one report, which used the same study participants as our present study, the OR was 23.96 (2.55–225.2) for the study participants who had cumulative arsenic exposure of more than 20 mg/liter × year and MMA percentage of more than 26.7% [28]. In addition, Yu et al. showed that the OR in the group with MMA percentage higher than 15.5% was 5.52 (1.22–24.81), as compared to the group with MMA percentage of less than 15.5% [29]. Nevertheless, our data presented no significance when grouped by 26.7% and 15.5%, and the ORs were 1.10 (0.51–2.37) and 1.43 (0.79–2.61), respectively (data not shown). However, when we grouped the participants by 13.81%, which was the median value of the control group, the OR in the high MMA percentage group became 2.01 (1.09–3.73) and as a result showed significance.

The polymorphisms analyzed in this study have been investigated for their roles in various diseases. In the GST group, the GSTM1 subtype was reported to correlate with cervical neoplasia and chronic obstructive pulmonary disease [30, 31], while GSTT1 subtype had a higher incidence or percentage of bladder cancer [32] and the GSTP1 Ile105Val polymorphism was related with squamous cell carcinoma (SCC) and colorectal cancer [33, 34]. NQO1 C609T was reported in many diseases, including lung cancer, bladder cancer, and esophageal squamous cell carcinoma [33, 35, 36]. Both EPHX1 polymorphisms in Tyr113His and His139Arg revealed significantly high risks in developing lung cancer and polycystic ovary syndrome [37, 38]. Furthermore, the correlation between the long HO-1 (GT) repeat and oral squamous cell carcinoma has been identified [39]. Among DNA repair genes, XRCC1 Arg399Gln has been reported in breast cancer and skin cancer [16, 40], while XPD C156A was correlated with basal cell carcinoma [41], and the hOGG1 Ser326Cys polymorphism has been studied as a causal factor in lung and esophageal cancers [20, 42, 43].

In the univariate analysis, GSTT1 genotype, EPHX1 Tyr113His, and XPD C156A polymorphisms showed significant differences between skin cancer patients and controls. After considering age, gender, cumulative arsenic exposure, and MMA percentage, none of the SNPs showed significant association with the disease risk. We suggest that this may be because the effect of arsenic was too strong and, therefore, the genetic effect was overshadowed after adjustments were made for two arsenic-correlated parameters. Gene-gene interactions conducted in different pathways showed different results. In the xenobiotic metabolic genes GSTs, there was a significant increase in equal to or more than 1-risk-polymorphism groups when compared to the 0-risk group, with a *P* value of 0.0074. These results indicated that the GST polymorphisms may exert strong effects in arsenic-induced skin cancer. ROS-related gene polymorphisms showed no significant differences in the 2- and 3-polymorphism groups compared to the group with less than 2 polymorphisms. On the other hand, a higher risk polymorphism number of DNA repair genes were associated with an increased disease risk, and test for trend was statistically significant. When considering GST family genes, EPHX1 Tyr113His and XPD C156A together, we found that the higher the polymorphism number among these genes, the higher the risk for skin cancer development. Test for trend was also statistically significant even considering multiple comparison issue. Compared to the referent group with less than 2 polymorphisms, the 2-polymorphism group did not show any significant differences, though the 3-polymorphism group showed significant increase in OR (4.67). These results indicated that participants who had 3 polymorphisms would have higher risks than the group with less than 1 polymorphism in developing arsenic-induced skin cancer.

Although we identified some changes in the gene-gene interaction analysis, there was no satisfactorily significant increase in OR values in the present study. Possible explanations may include the following: (i) carcinogenesis is known to be a multistep process, and perhaps any one or few of these genes were not sufficient in their effects in tumor formation; (ii) the polymorphisms were chosen from previous studies that reportedly correlated with diseases, but there might be alternative or additional genes that are more crucial in the development of arsenic-induced skin cancer. For example, when considering the association of ROS genes with skin cancer, we did not include catalase or superoxide dismutase in the present study; (iii) many of the genes in this study belonged to different pathways involved in cellular activities and therefore their corresponding effects and impact might not be in concert. Another potential issue was the inherent limitation in the sample size, which only included a study population of 280 participants. It appears that this sample size was insufficient, especially during analysis of gene-gene interactions, where we were compelled to combine two or three groups to calculate the OR values because case numbers were too few in some groups. Furthermore, we were unfortunately unable to obtain all available data on cumulative arsenic exposure and MMA percentage, as the possession of complete information would no doubt influence the results in adjustment and further analysis.

In conclusion, we suggest that GSTT1, EPHX1 Tyr113His, and XPD C156A polymorphisms are potential genetic factors for arsenic-induced skin carcinogenesis. Individuals who carried all the 3 risk polymorphisms, including any risk variant of GSTs, EPHX1 Tyr113His, and XPD C156A polymorphisms, had a 4.86-fold risk for the development of arsenic-related skin cancers compared to those with less than 2 polymorphisms. Genes that are even more critical and are involved in arsenic-induced skin cancers remain to be identified and investigated.

## Figures and Tables

**Table 1:** PCR primers and restriction enzymes of selected polymorphisms.

Gene	Primer	Enzyme
*GSTM1 *	Forward: GAACTCCCTGAAAAGCTAAAGC	
	Reverse: GTTGGGCTCAAATATACGGTGG	

*GSTT1 *	Forward: TTCCTTACTGGTCCTCACATCTC	
	Reverse: CAGCTGCATTTGGAAGTGCTC	

*GSTP1* (Ile105Val, rs1695)	Forward: GTAGTTTGCCCAAGGTCAAG	BsmAI
	Reverse: AGCCACCTGAGGGGTAAG	

*NQO1* (C609T, rs1800566)	Forward: AAGCCCAGACCAACTTCT	HinfI
	Reverse: ATTTGAATTCGGGCGTCTGCTG	

*EPHX1* (Tyr113His, rs1051740)	Forward: CTTGAGCTCTGTCCTTCCCATCCC	Tth111I
	Reverse: AATCTTAGTCTTGAAGTGACGGT	

*EPHX1* (His139Arg, rs2234922)	Forward: ACATCCACTTCATCCACGT	Rsa1
	Reverse: ATGCCTCTGAGAAGCCAT	

*HO-1* (5′-site GT repeats)	Forward: FAM-AGAGCCTGCAGCTTCTCAGA	
	Reverse: ACAAAGTCTGGCCATAGGAC	

*XRCC1* (Arg280His, rs25489)	Forward: GGCTGGGACCACCTGTGTT	RsaI
	Reverse: TTGACCCCCAGTGGTGCTAA	

*XRCC1* (Arg399Gln, rs25487)	Forward: TCTCCCTTGGTCTCCAACCT	MspI
	Reverse: AGTAGTCTGCTGGCTCTGG	

*XPD* (C156A, rs238406)	Forward: TGGAGTGCTATGGCACGATCTCT	TflI
	Reverse: CCATGGGCATCAAATTCCTGGGA	

*hOGG1* (Ser326Cys, rs1052133)	Forward: ACTGTCACTAGTCTCACCAG	Fnu4HI
	Reverse: TGAATTCGGAAGGTGCTTGGGGAAT	

*ATM* (INV48 +238C>G, rs609429)	Forward: CCTGGTTATAAAATGAGAAGG	KpnI
	Reverse: GCAGCAACTACCATTCATTGAG	

**Table 2:** Distribution of demographic variables, arsenic exposure level, and percentage of monomethylarsonic acid (MMA) between skin cancer cases and age and sex matched controls.

Variable	Group	Case		Control		OR (95% CI)	
		Number	(%)	Number	(%)		
Age	(Mean ± Std deviation)	56.13 ± 6.87		54.38 ± 6.26			

Gender	Male	46	(65.71)	138	(65.71)		
	Female	24	(34.29)	72	(34.29)		

Cumulative as exposure (ppm-year)	<10.0	4	(5.71)	40	(19.05)	1.00	(Referent)
	10.0–19.9	22	(31.43)	62	(29.52)	3.55	(1.14–11.06)
	20.0+	31	(44.29)	59	(28.10)	5.25	(1.72–16.05)
	Unknown	13	(18.57)	49	(23.33)	2.65	(0.80–8.77)

MMA percentage (%)	0–13.81	21	(30.00)	94	(44.76)	1.00	(Referent)
	13.81+	36	(51.43)	80	(38.10)	2.01	(1.09–3.73)
	Unknown	13	(18.57)	36	(17.14)	1.61	(0.73–3.57)

**Table 3:** Univariate analysis of genetic polymorphisms in glutathione S-transferase family, reactive oxidative stress genes, and DNA repair genes.

Gene	Case		Control		OR (95% CI)				*P* value
	Number	(%)	Number	(%)					
*GSTM1 *									
Nonnull	25	(35.71)	86	(41.15)	1.00	(Referent)			0.42
Null	45	(64.29)	123	(58.85)	1.26	(0.72–2.21)			

*GSTT1 *									
Nonnull	27	(38.57)	109	(52.15)	1.00	(Referent)			0.05
Null	43	(61.43)	100	(47.85)	1.74	(1.00–3.02)			

*GSTP1 *(Ile105Val, rs1695)									
W/W	44	(62.86)	143	(68.10)	1.00	(Referent)	1.00	(Referent)	0.42
W/M	26	(37.14)	59	(28.10)	1.47	(0.83–2.61)	1.26	(0.72–2.22)	
M/M	0	—	8	(3.81)	—	—			

*NQO1 *(C609T, rs1800566)									
C/C	18	(26.47)	70	(35.00)	1.00	(Referent)	1.00	(Referent)	0.19
C/T	36	(52.94)	104	(52.00)	1.35	(0.71–2.56)	1.50	(0.81–2.76)	
T/T	14	(20.59)	26	(13.00)	2.09	(0.91–4.81)			

*EPHX1 *(Tyr113His, rs1051740)									
T/T	4	(6.15)	31	(16.40)	1.00	(Referent)	1.00	(Referent)	0.04
T/C	27	(41.54)	56	(29.63)	3.74	(1.20–11.66)	2.99	(1.01–8.83)	
C/C	34	(52.31)	102	(53.97)	2.58	(0.85–7.85)			

*EPHX1* (His139Arg, rs2234922)									
G/G	0	—	4	(1.94)	1.00	(Referent)			0.49
A/G	15	(22.06)	50	(24.27)					
A/A	53	(77.94)	152	(73.79)	0.80	(0.42–1.53)			

*HO-1* (5′-site GT repeats)									
L/L	17	(26.15)	47	(23.62)	1.00	(Referent)			0.87
S/L	34	(52.31)	111	(55.78)					
S/S	14	(21.54)	41	(20.60)	1.06	(0.53–2.10)			

*XRCC1* (Arg280His, rs25489)									
His/His	0	—	4	(1.93)	1.00	(Referent)			0.16
Arg/His	9	(13.04)	39	(18.84)					
Arg/Arg	60	(86.96)	164	(79.23)	1.75	(0.80–3.80)			

*XRCC1* (Arg399Gln, rs25487)									
Arg/Arg	35	(50.72)	114	(55.34)	1.00	(Referent)	1.00	(Referent)	0.50
Arg/Gln	26	(37.68)	71	(34.47)	1.19	(0.66–2.15)	1.20	(0.70–2.08)	
Gln/Gln	8	(11.59)	21	(10.19)	1.24	(0.51–3.05)			

*XPD* (C156A, rs238406)									
CC	10	(14.93)	54	(26.34)	1.00	(Referent)	1.00	(Referent)	0.05
AC	40	(59.70)	110	(53.66)	1.96	(0.91–4.22)	2.04	(0.99–4.27)	
AA	17	(25.37)	41	(20.00)	2.24	(0.93–5.40)			

*hOGG1* (Ser326Cys, rs1052133)									
Cys/Cys	22	(32.35)	82	(39.42)	1.00	(Referent)	1.00	(Referent)	0.30
Ser/Cys	37	(54.41)	103	(49.52)	1.34	(0.73–2.45)	1.36	(0.76–2.43)	
Ser/Ser	9	(13.24)	23	(11.06)	1.46	(0.59–3.60)			

*ATM* (INV48 +238C>G, rs609429)									
CC	21	(36.21)	77	(37.75)	1.00	(Referent)	1.00	(Referent)	0.84
CG	26	(44.83)	89	(43.63)	1.07	(0.56–2.05)	1.07	(0.58–1.95)	
GG	11	(18.97)	38	(18.63)	1.06	(0.46–2.42)			

**Table 4:** Multivariate analysis of genetic polymorphisms in glutathione S-transferase family, reactive oxidative stress genes, and DNA repair genes.

Gene	Case		Control		OR (95% CI)				*P* value
	Number	(%)	Number	(%)					
*GSTM1 *									
Nonnull	25	(35.71)	86	(41.15)	1.00	(Referent)			0.46
Null	45	(64.29)	123	(58.85)	1.26	(0.68–2.39)			

*GSTT1 *									
Nonnull	27	(38.57)	109	(52.15)	1.00	(Referent)			0.30
Null	43	(61.43)	100	(47.85)	1.36	(0.76–2.46)			

*GSTP1 *(Ile105Val, rs1695)									
W/W	44	(62.86)	143	(68.10)	1.00	(Referent)	1.00	(Referent)	0.56
W/M	26	(37.14)	59	(28.10)	1.17	(0.57–2.37)	1.21	(0.64–2.30)	
M/M	0	—	8	(3.81)	—	—			

*NQO1 *(C609T, rs1800566)									
C/C	18	(26.47)	70	(35.00)	1.00	(Referent)	1.00	(Referent)	0.41
C/T	36	(52.94)	104	(52.00)	1.08	(0.51–2.29)	1.33	(0.68–2.58)	
T/T	14	(20.59)	26	(13.00)	2.12	(0.77–5.86)			

*EPHX1 *(Tyr113His, rs1051740)									
T/T	4	(6.15)	31	(16.40)	1.00	(Referent)	1.00	(Referent)	0.07
T/C	27	(41.54)	56	(29.63)	3.25	(1.01–10.54)	2.71	(0.90–8.25)	
C/C	34	(52.31)	102	(53.97)	2.32	(0.74–7.31)			

*EPHX1* (His139Arg, rs2234922)									
G/G	0	—	4	(1.94)	1.00	(Referent)			0.35
A/G	15	(22.06)	50	(24.27)					
A/A	53	(77.94)	152	(73.79)	0.71	(0.35–1.48)			

*HO-1* (5′-site GT repeats)									
L/L	17	(26.15)	47	(23.62)	1.00	(Referent)			0.45
S/L	34	(52.31)	111	(55.78)					
S/S	14	(21.54)	41	(20.60)	0.94	(0.42–2.08)			

*XRCC1* (Arg280His, rs25489)									
His/His	0	—	4	(1.93)	1.00	(Referent)			0.39
Arg/His	9	(13.04)	39	(18.84)					
Arg/Arg	60	(86.96)	164	(79.23)	0.69	(0.28–1.62)			

*XRCC1* (Arg399Gln, rs25487)									
Arg/Arg	35	(50.72)	114	(55.34)	1.00	(Referent)	1.00	(Referent)	0.76
Arg/Gln	26	(37.68)	71	(34.47)	1.18	(0.57–2.43)	1.11	(0.59–2.07)	
Gln/Gln	8	(11.59)	21	(10.19)	0.90	(0.30–2.66)			

*XPD* (C156A, rs238406)									
CC	10	(14.93)	54	(26.34)	1.00	(Referent)	1.00	(Referent)	0.17
AC	40	(59.70)	110	(53.66)	1.60	(0.72–3.55)	1.71	(0.79–3.69)	
AA	17	(25.37)	41	(20.00)	2.06	(0.82–5.19)			

*hOGG1* (Ser326Cys, rs1052133)									
Cys/Cys	22	(32.35)	82	(39.42)	1.00	(Referent)	1.00	(Referent)	0.41
Ser/Cys	37	(54.41)	103	(49.52)	1.16	(0.57–2.37)	0.77	(0.41–1.45)	
Ser/Ser	9	(13.24)	23	(11.06)	1.17	(0.38–3.63)			

*ATM* (INV48 +238C>G, rs609429)									
CC	21	(36.21)	77	(37.75)	1.00	(Referent)	1.00	(Referent)	0.81
CG	26	(44.83)	89	(43.63)	1.08	(0.49–2.34)	1.08	(0.57–2.08)	
GG	11	(18.97)	38	(18.63)	1.44	(0.57–3.69)			

OR adjusted for age, gender, cumulative arsenic exposure, and monomethylarsonic acid (MMA) percentage.

**Table 5:** Combination analysis of the polymorphisms with skin cancers in the groups of xenobiotic metabolic genes, reactive oxidative stress genes, DNA repair genes, and three highly risk polymorphisms.

Genotype	Case		Control		Multivariate-adjusted		*P* value for trend
	Number	(%)	Number	(%)	OR (95% CI)		
Xenobiotic metabolic genes							
Wild types: *GSTM1* nonnull, *GSTT1* nonnull, *GSTP1* (Ile105Val) W/W							
0 polymorphisms	4	(5.71)	43	(20.57)	1.00	(Referent)	0.0074
1 polymorphism	27	(38.57)	69	(33.01)	4.27	(1.48–12.38)	
2 polymorphisms	30	(42.86)	70	(33.50)			
3 polymorphisms	9	(12.86)	27	(12.92)			

Reactive oxidative stress genes							
Wild types: *NQO1* (C609T) C/C, *EPHX* (Tyr113His) T/T, *HO-1 *(5′-site GT repeats) L/L & S/L							
0 polymorphisms	1	(1.59)	7	(4.02)	1.00	(Referent)	0.12
1 polymorphism	15	(23.81)	57	(32.76)			
2 polymorphisms	39	(61.90)	93	(53.45)	1.68	(0.86–3.26)	
3 polymorphisms	8	(12.70)	17	(9.77)	1.88	(0.69–5.13)	

*DNA repair genes*							
Wild types: *XRCC1* (Arg280His) His/His & His/Arg, *XPD* (C156A) C/C, *hOGG1* (Ser326Cys) Cys/Cys, *ATM* (INV48 +238C>G) C/C * *							
0 polymorphisms	1	(1.85)	1	(0.51)	1.00	(Referent)	0.0334
1 polymorphism	0	—	16	(8.16)			
2 polymorphisms	15	(27.78)	60	(30.61)			
3 polymorphisms	20	(37.04)	75	(38.27)	4.40	(0.57–34.26)	
4 polymorphisms	18	(33.33)	44	(22.45)	6.95	(0.86–56.23)	

Three highly risk polymorphisms							
Wild types: no risk polymorphism of the *GST*s (*GSTM1* nonnull, *GSTT1* nonnull, *GSTP1* W/W), *EPHX1* (Tyr113His) T/T, *XPD* (C156A) * *							
C/C							
0 polymorphisms	0	—	2	(1.08)	1.00	(Referent)	0.0004
1 polymorphism	2	(3.17)	16	(8.60)			
2 polymorphisms	14	(22.22)	81	(43.55)	1.56	(0.32–7.45)	
3 polymorphisms	47	(74.60)	87	(46.77)	4.86	(1.08–21.85)	

ROS: reactive oxidative stress.

## References

[1] Abernathy C. O., Thomas D. J., Calderon R. L. (2003). Health effects and risk assessment of arsenic. *Journal of Nutrition*.

[2] Chiou H.-Y., Hsueh Y.-M., Liaw K.-F. (1995). Incidence of internal cancers and ingested inorganic arsenic: a seven-year follow-up study in Taiwan. *Cancer Research*.

[3] Morales K. H., Ryan L., Kuo T.-L., Wu M.-M., Chen C.-J. (2000). Risk of internal cancers from arsenic in drinking water. *Environmental Health Perspectives*.

[4] Rossman T. G., Uddin A. N., Burns F. J. (2004). Evidence that arsenite acts as a cocarcinogen in skin cancer. *Toxicology and Applied Pharmacology*.

[5] Chen C.-J., Hsu L.-I., Wang C.-H. (2005). Biomarkers of exposure, effect, and susceptibility of arsenic-induced health hazards in Taiwan. *Toxicology and Applied Pharmacology*.

[6] Tseng W. P., Chu H. M., How S. W., Fong J. M., Lin C. S., Yeh S. (1968). Prevalence of skin cancer in an endemic area of chronic arsenicism in Taiwan. *Journal of the National Cancer Institute*.

[7] Kitchin K. T., Ahmad S. (2003). Oxidative stress as a possible mode of action for arsenic carcinogenesis. *Toxicology Letters*.

[8] Qian Y., Castranova V., Shi X. (2003). New perspectives in arsenic-induced cell signal transduction. *Journal of Inorganic Biochemistry*.

[9] Kitchin K. T. (2001). Recent advances in arsenic carcinogenesis: modes of action, animal model systems, and methylated arsenic metabolites. *Toxicology and Applied Pharmacology*.

[10] Wu M.-M., Chiou H.-Y., Lee T.-C. (2010). GT-repeat polymorphism in the heme oxygenase-1 gene promoter and the risk of carotid atherosclerosis related to arsenic exposure. *Journal of Biomedical Science*.

[11] Wu M. M., Chiou H. Y., Chen C. L. (2010). GT-repeat polymorphism in the heme oxygenase-1 gene promoter is associated with cardiovascular mortality risk in an arsenic-exposed population in northeastern Taiwan. *Toxicology and Applied Pharmacology*.

[12] Zhang L., Song F. F., Huang Y. B., Zheng H., Song F. J., Chen K. X. (2014). Association between the (GT)n polymorphism of the HO-1 gene promoter region and cancer risk: a meta-analysis. *Asian Pacific Journal of Cancer Prevention*.

[13] Lajin B., Alachkar A. (2013). The NQO1 polymorphism C609T (Pro187Ser) and cancer susceptibility: a comprehensive meta-analysis. *British Journal of Cancer*.

[14] Hassett C., Aicher L., Sidhu J. S., Omiecinski C. J. (1994). Human microsomal epoxide hydrolase: genetic polymorphism and functional expression *in vitro* of amino acid variants. *Human Molecular Genetics*.

[15] Han J., Hankinson S. E., Colditz G. A., Hunter D. J. (2004). Genetic variation in XRCC1, sun exposure, and risk of skin cancer. *British Journal of Cancer*.

[16] Nelson H. H., Kelsey K. T., Mott L. A., Karagas M. R. (2002). The XRCC1 Arg399Gln polymorphism, sunburn, and non-melanoma skin cancer: evidence of gene-environment interaction. *Cancer Research*.

[17] Zhang H., Li W., Franklin M. J., Dudek A. Z. (2011). Polymorphisms in DNA repair gene XRCC1 and skin cancer risk: a meta-analysis. *Anticancer Research*.

[18] Bravard A., Vacher M., Moritz E. (2009). Oxidation status of human OGG1-S326C polymorphic variant determines cellular DNA repair capacity. *Cancer Research*.

[19] Le Marchand L., Donlon T., Lum-Jones A., Seifried A., Wilkens L. R. (2002). Association of the hOGG1 Ser326Cys polymorphism with lung cancer risk. *Cancer Epidemiology Biomarkers and Prevention*.

[20] Xing D. Y., Tan W., Song N., Lin D. X. (2001). Ser326Cys polymorphism in hOGG1 gene and risk of esophageal cancer in a Chinese population. *International Journal of Cancer*.

[21] Ma L., Chu H., Wang M. (2012). hOGG1 Ser326Cys polymorphism is associated with risk of bladder cancer in a Chinese population: a case-control study. *Cancer Science*.

[22] Coin F., Marinoni J.-C., Rodolfo C., Fribourg S., Pedrini A. M., Egly J.-M. (1998). Mutations in the XPD helicase gene result in XP and TTD phenotypes, preventing interaction between XPD and the p44 subunit of TFIIH. *Nature Genetics*.

[23] Vogel U., Olsen A., Wallin H., Overvad K., Tjønneland A., Nexø B. A. (2005). Effect of polymorphisms in XPD, RAI, ASE-1 and ERCC1 on the risk of basal cell carcinoma among Caucasians after age 50. *Cancer Detection and Prevention*.

[24] Lovatt T., Alldersea J., Lear J. T. (2005). Polymorphism in the nuclear excision repair gene ERCC2/XPD: association between an exon 6-exon 10 haplotype and susceptibility to cutaneous basal cell carcinoma. *Human Mutation*.

[25] Appelbaum K. M., Karagas M. R., Hunter D. J. (2007). Polymorphisms in nucleotide excision repair genes, arsenic exposure, and non-melanoma skin cancer in New Hampshire. *Environmental Health Perspectives*.

[26] Chen Y.-C., Guo Y.-L. L., Su H.-J. J. (2003). Arsenic methylation and skin cancer risk in Southwestern Taiwan. *Journal of Occupational and Environmental Medicine*.

[27] Hsueh Y.-M., Cheng G.-S., Wu M.-M., Yu H.-S., Kuo T.-L., Chen C.-J. (1995). Multiple risk factors associated with arsenic-induced skin cancer: effects of chronic liver disease and malnutritional status. *British Journal of Cancer*.

[28] Hsueh Y.-M., Chiou H.-Y., Huang Y.-L. (1997). Serum *β*-carotene level, arsenic methylation capability, and incidence of skin cancer. *Cancer Epidemiology Biomarkers and Prevention*.

[29] Yu R. C., Hsu K.-H., Chen C.-J., Froines J. R. (2000). Arsenic methylation capacity and skin cancer. *Cancer Epidemiology Biomarkers & Prevention*.

[30] Sierra-Torres C. H., Au W. W., Arrastia C. D. (2003). Polymorphisms for chemical metabolizing genes and risk for cervical neoplasia. *Environmental and Molecular Mutagenesis*.

[31] Cheng S. L., Yu C. J., Chen C. J., Yang P. C. (2004). Genetic polymorphism of epoxide hydrolase and glutathione S-transferase in COPD. *European Respiratory Journal*.

[32] Sanyal S., Festa F., Sakano S. (2004). Polymorphisms in DNA repair and metabolic genes in bladder cancer. *Carcinogenesis*.

[33] Lin P., Hsueh Y.-M., Ko J.-L., Liang Y.-F., Tsai K.-J., Chen C.-Y. (2003). Analysis of NQO1, GSTP1, and MnSOD genetic polymorphisms on lung cancer risk in Taiwan. *Lung Cancer*.

[34] Stoehlmacher J., Park D. J., Zhang W. (2004). A multivariate analysis of genomic polymorphisms: prediction of clinical outcome to 5-FU/oxaliplatin combination chemotherapy in refractory colorectal cancer. *British Journal of Cancer*.

[35] Park S.-J., Zhao H., Spitz M. R., Grossman H. B., Wu X. (2003). An association between NQO1 genetic polymorphism and risk of bladder cancer. *Mutation Research*.

[36] Zhang J., Schulz W. A., Li Y. (2003). Association of NAD(P)H: quinone oxidoreductase 1 (NQO1) C609T polymorphism with esophageal squamous cell carcinoma in a German Caucasian and a northern Chinese population. *Carcinogenesis*.

[37] Gsur A., Zidek T., Schnattinger K. (2003). Association of microsomal epoxide hydrolase polymorphisms and lung cancer risk. *British Journal of Cancer*.

[38] Korhonen S., Romppanen E.-L., Hiltunen M. (2003). Two exonic single nucleotide polymorphisms in the microsomal epoxide hydrolase gene are associated with polycystic ovary syndrome. *Fertility and Sterility*.

[39] Chang K.-W., Lee T.-C., Yeh W.-I. (2004). Polymorphism in *heme oxygenase-1* (*HO-1*) promoter is related to the risk of oral squamous cell carcinoma occurring on male areca chewers. *British Journal of Cancer*.

[40] Duell E. J., Millikan R. C., Pittman G. S. (2001). Polymorphisms in the DNA repair gene XRCC1 and breast cancer. *Cancer Epidemiology Biomarkers and Prevention*.

[41] Dybdahl M., Vogel U., Frentz G., Wallin H., Nexø B. A. (1999). Polymorphisms in the DNA repair gene XPD: correlations with risk and age at onset of basal cell carcinoma. *Cancer Epidemiology Biomarkers and Prevention*.

[42] Sugimura H., Kohno T., Wakai K. (1999). hOGG1 Ser326Cys polymorphism and lung cancer susceptibility. *Cancer Epidemiology Biomarkers and Prevention*.

[43] Wikman H., Risch A., Klimek F. (2000). hOGG1 polymorphism and loss of heterozygosity (LOH): significance for lung cancer susceptibility in a caucasian population. *International Journal of Cancer*.

